# Transcriptomic clock predicts vascular changes of prodromal diabetic retinopathy

**DOI:** 10.1038/s41598-023-40328-w

**Published:** 2023-08-10

**Authors:** Huishi Toh, Alexander Smolentsev, Ryan Sadjadi, Dennis Clegg, Jingqi Yan, Ron Stewart, James A. Thomson, Peng Jiang

**Affiliations:** 1 Neuroscience Research Institute, University of California Santa Barbara, Santa Barbara, CA USA; 2 Department of Molecular, Cellular and Developmental Biology, University of California Santa Barbara, Santa Barbara, CA USA; 3 Morgridge Institute For Research, Madison, WI 53706 USA; 4 Department of Biological, Geological and Environmental Sciences, Cleveland State University, Cleveland, OH 44115 USA; 5 Center for Gene Regulation in Health and Disease, Cleveland State University, Cleveland, OH 44115 USA; 6 Center for RNA Science and Therapeutics, School of Medicine, Case Western Reserve University, 10900 Euclid Avenue, Cleveland, OH 44106 USA

**Keywords:** Computational biology and bioinformatics, Computational models, Data mining, Machine learning, Molecular biology, Transcriptomics, Molecular medicine, Predictive markers

## Abstract

Diabetic retinopathy is a common complication of long-term diabetes and that could lead to vision loss. Unfortunately, early diabetic retinopathy remains poorly understood. There is no effective way to prevent or treat early diabetic retinopathy until patients develop later stages of diabetic retinopathy. Elevated acellular capillary density is considered a reliable quantitative trait present in the early development of retinopathy. Hence, in this study, we interrogated whole retinal vascular transcriptomic changes via a Nile rat model to better understand the early pathogenesis of diabetic retinopathy. We uncovered the complexity of associations between acellular capillary density and the joint factors of blood glucose, diet, and sex, which was modeled through a Bayesian network. Using segmented regressions, we have identified different gene expression patterns and enriched Gene Ontology (GO) terms associated with acellular capillary density increasing. We developed a random forest regression model based on expression patterns of 14 genes to predict the acellular capillary density. Since acellular capillary density is a reliable quantitative trait in early diabetic retinopathy, and thus our model can be used as a transcriptomic clock to measure the severity of the progression of early retinopathy. We also identified NVP-TAE684, geldanamycin, and NVP-AUY922 as the top three potential drugs which can potentially attenuate the early DR. Although we need more in vivo studies in the future to support our re-purposed drugs, we have provided a data-driven approach to drug discovery.

## Introduction

Diabetic retinopathy is a common diabetes complication of long-term diabetes that results in damage to the retinal blood vessels and can progress towards vision loss. In several developed countries, including the United States, diabetic retinopathy is the leading cause of new cases of blindness among adults aged 20 to 74^1^. People living with diabetes, regardless of clinical subtypes, have roughly one-third of a chance to develop diabetic retinopathy^2^, similarly affecting both males and females in general^3,4^.

At the early stages, non-proliferative diabetic retinopathy (DR) can be detected by screening when vision is not yet affected. However, there is no effective way to slow down or stop the progression of the disease to prevent vision loss, where patients are frequently left untreated until later stages. Moreover, diabetic retinopathy is not reversible and will continue to progress. Eventually, some cases will develop into proliferative diabetic retinopathy, where fibrosis develops on the retinal surface, and abnormal hemorrhage-prone new vessels lead to vision loss^5^. To prevent vision loss from diabetic retinopathy, we need to understand the early pathogenesis of asymptomatic retinopathy. Unfortunately, these early processes remain poorly understood partly due to insufficient histological material from human donors and limited animal models which can recapitulate the natural progression of human diabetic retinopathy^6^.

Nile rat (*Arvicanthis niloticus*), native to Northern Africa, is a diurnal rodent with a general diet of grass stems^7^. However, when fed a conventional rodent chow in a laboratory environment, the Nile rat quickly develops diet-induced diabetes in both sexes. To clarify, conventional rodent chow, though meant to sustain healthy common laboratory rodents, is hypercaloric, relative to the native diet, for the Nile rat species and will be thereafter described as a high-caloric diet. To prevent diet-induced diabetes in a laboratory environment, the Nile rat can be fed a high-fiber diet^8^. Thus, diet-induced diabetes in the Nile rat appear to follow the natural progression of type 2 diabetes in humans. Our previous study demonstrated that diabetic Nile rats could develop retinopathy with advanced retinal lesions similar to clinical features linked to vision loss in humans, such as macular edema, capillary non-perfusion, and proliferative diabetic retinopathy^9^. Specifically, Nile rat lesions included retinal leakage, retinal thickening, capillary non-perfusion, intraretinal microvascular abnormalities, and apparent proliferative diabetic retinopathy, based on observed neovascularization beyond the retinal surface^9^. Typically, other rodent models with diet-induced diabetic retinopathy do not exhibit the exact microvascular damage seen in humans and often lack proliferative neovascularization. However, neovascularization was observed in relatively older Nile rats and age might play a role in neovascularization. In that study, we also demonstrated that elevated acellular capillary density is a reliable quantitative trait present in the initial development of retinopathy, preceding additional vascular dysfunction in pericytes and endothelial cells^9^. The use of retinal acellular capillary density as one of the earliest markers of retinopathy is consistent in both Nile rat^9^ and in humans^10^. Therefore, we reasoned that retinal vasculature change is an important causal factor in initiating and driving the pathogenesis of diabetic retinopathy. Since retinal acellular capillary density is one of the initial detectable changes relevant to retinal vascular dysfunction, we could use acellular capillary density to mark early disease progression in diabetic retinopathy.

In this study, we look for gene signatures that correspond to the progression of increasing acellular capillary density in the vascular network of the whole retina. We performed RNA-Seq on freshly isolated retinal vascular tissue from 28 male and female Nile rats on either a high-caloric or high-fiber diet. We knew from our previous study that the acellular capillary density is a bilateral phenotype where left and right eyes are highly correlated within individual Nile rats^9^. Taking advantage of the bilateral nature of this phenotype, we could use one eye to determine the acellular capillary density that required fixed tissue and the contralateral eye to harvest fresh tissue for RNA sequencing. We identified gene signatures associated with increasing acellular capillary density, a quantitative marker of early retinopathy. Using a random forest machine learning model, we identified a combination of fourteen genes that can quantitatively predict acellular capillary density that can serve as a vascular-specific transcriptomic clock denoting the progression of retinopathy.

Leveraging a large-scale chemical compound/drug perturbated gene expression signature database (LINCS L1000), we identified NVP-TAE684, geldanamycin, and NVP-AUY922 as the top three potential drugs which can reverse the acellular capillary density correlated genes signatures, thereby providing putative drug candidates to address the early stages of retinopathy. Interestingly, both geldanamycin and NVP-AUY922 are Hsp90 inhibitors^11,12^. Studies have shown that Hsp90 inhibitors can prevent retinal degeneration in models of retinitis pigmentosa and age-related macular degeneration^13^. Although our re-purposed candidate drugs require further validation in vivo, we have provided a novel data-driven approach to prioritize compounds that can potentially block the progression of retinopathy prior to permanent vision impairment. Our study also reveals retinal vascular transcriptomics associated with the early pathogenesis of retinopathy.

## Methods

### Animal protocol and ethical statement

All animal experiments were approved by the University of California Santa Barbara, Institutional Animal Care and Use Committee, and conducted in accordance with the NIH Guide for the Care and Use of Laboratory Animals; study protocol 893. UCSB founder Nile rats were derived from the Brandeis University colony of the KC Hayes Laboratory. Nile rats in UCSB are housed in a 12-h light cycle (10 a.m. to 10 p.m.) room at 21–26 °C in a conventional facility with individually ventilated cages and are provided autoclaved Sanichips as bedding material, with additional cage enrichment, including wooden blocks and plastic bones. Insulin was not administered to the diabetic rats. To avoid serious adverse effects from diabetes, we euthanized all animals with over 5% weight loss or a large drop of over 100 mg/dL in RBG. To identify these animals, we do a weekly check of the weight and RBG of all Nile rats after they reached RBG ≥ 500 mg/dL.

Nile rats are either fed a high-fiber diet (Lab Diet 5L3M; Newco Speciality, Rancho Cucamonga, CA, USA) or a high-caloric diet (Formulab Diet 5008; Newco Speciality, Rancho Cucamonga, CA, USA)^14^. The percentage of crude fiber is 23% for the high-fiber diet and only 4% for the high-caloric diet. The ratio for the percentage of calories provided by carbohydrate, fat, and protein were 67:10:23 for the high-fiber diet and 56:17:27 for the high-caloric diet. Random blood glucose (RBG) levels are measured using a glucometer (Contour) every four weeks (between 2 and 5 p.m.) starting at weaning age (4 weeks old) with blood from a tail prick. There were 32 Nile rats in total involved in this study. Four out of thirty-two Nile rat samples failed at the RNA-seq library step (whole retinal vasculature). Thus, we have 28 Nile rats in total for RNA-seq data analysis. The detailed Nile rat information, such as acellular capillary density, average random blood glucose, duration of diabetes, sex, age, diet, and whether it is included in the RNA-seq data analysis, is shown in Supplemental Table 1. All animals are monitored for signs of development of diabetic complications that would be considered distressful to the animal, which include but are not limited to dehydration (severe polydipsia and polyuria, an indication of kidney failure), or pronounced and sustained weakness or lethargy, when we will apply the humane endpoint to these distressed animals. Euthanasia is carried out by carbon dioxide inhalation. This study is reported in accordance with ARRIVE guidelines (https://arriveguidelines.org).

### Retinal trypsin digest and sample preparation of retinal vasculature for RNA sequencing

From each animal, we did a trypsin digest on one retina and collected the retinal vasculature for RNA-Seq from the other retina. The retinal vasculature was loosened from other retinal cells using osmotic lysis, where the retina was placed in RNase-free water for 1 h at 4 °C on a shaker. Next, water was aspirated, and the remaining retinal tissue was incubated in a 10% DNase solution at 37 °C for 15 min. Then, the retina was transferred to a petri-dish with RNase-free water. Working under a dissecting microscope, we used a P200 pipet to squirt water on the retina until the inner limiting membrane was detached and neuronal cells were washed away from the retinal vasculature. Finally, the resulting retinal vasculature is preserved in RNAlater overnight at 4 °C and stored at − 80 °C. For the trypsin digest, we used our previously published method^9^.

### RNA-seq of retinal vasculature

Total RNAs from 32 Nile rat vascular samples were isolated using trizol (ThermoFisher #15596018) and chloroform phase separations followed by the RNeasy mini protocol (Qiagen #74106) with optional on-column DNase digestion (Qiagen #79254). One hundred nanograms of total RNA was used to prepare sequencing libraries using the LM-Seq (Ligation Mediated Sequencing) protocol^15^. RNAs were selected using the NEB Next Poly A + Isolation Kit (NEB #E7490S/L). Poly A + fractions were eluted, primed, and fragmented for 7 min at 85 °C. First stand cDNA synthesis was performed using SmartScribe Reverse Transcriptase (Takara Bio USA #639538), and RNA was removed. cDNA fragments were purified with AMpure XP beads (Beckman Coulter #A63881). The 5′ adapter was ligated, and 18 cycles of amplification were performed. These final indexed cDNA libraries were quantified, normalized, multiplexed, and run as single-end reads on the HiSeq 3000 sequencer. Four out of thirty-two Nile rat RNA-seq samples were failed during the library preparation step, and thus were excluded in the RNA-seq data analysis. Hence, there are 28 Nile rat RNA-seq samples in the downstream data analysis.

### RNA-seq data analysis

We used our lab-developed CRSP tool^16^ to estimate the gene expression values of Nile rat retina vascular tissues. Specifically, the retinal vasculature RNA-seq reads were mapped to our lab pre-built Nile rat transcriptomic contigs, assembled via Trinity tool^17^ based on ultra-deep sequencing (2.3 billion × 2 paired-end reads) of a pooled 22 major Nile rat organs^16^. The transcriptomic contigs were annotated based on mouse proteins via NCBI BLASTX tool^18^ with E-value < 10^–5^. A contig level abundance estimation is performed via RSEM^19^. For each Nile grass rat retina vascular RNA-seq sample, gene-level RNA-seq mapped read counts are obtained by summing the counts of the contigs that are mapped to proteins with the same gene symbol. The relative abundances, in terms of transcripts per million (TPM), for genes were computed by first normalizing each gene’s fragment count by the sum of the “effective lengths” (length less the read length) of the contigs mapped to that gene and then scaling the resulting values such that they sum to one million over all genes. The TPM values were further normalized via the median-of-ratio method by EBSeq^20^ R package.

### Retina acellular capillary count reading

Micrographs for quantification were taken with a Canon Rebel XSi digital camera (Canon, Tokyo, Japan) attached to an Olympus CKX41 microscope via an LM scope C-mount. For acellular capillary counts, 12 micrographs were taken from 3 randomly selected areas in each of the 4 retinal quadrants and analyzed at a final magnification of ×200. We categorized abnormal capillary features into three groups: the first group is definitive acellular capillaries; the second group is pericyte processes (described in our paper^9^) and the third group is ambiguous abnormal features that were very short or sometimes partially obscured by other vessels. The total area used for quantification corresponded to approximately 8.5% of the whole retinal area. The counts were quantified using FIJI computer software without any additional plugins by two masked graders.

### Segmented regression

To detect gene expression patterns associated with acellular capillary density levels, we performed a segmented regression analysis. Segmented regression is a method in which the independent variable is partitioned into intervals and a separate linear regression to fit each interval. For each gene, the number of breakpoints was determined by the lowest Bayesian Information Criterion (BIC)^21^ via enumerating all K (K <  = N) a possible number of breakpoints, where N was the number of time points. An optimal model will be found for every gene. We further defined the goodness of fit of the optimal model as the adjusted correlation coefficient R^2^:

$${R}^{2}=1-(1- {\overline{R} }^{2})\frac{N-1}{N-\left(\overline{K }+1\right)-1}$$

where is the optimally chosen K for a specific gene and N is the number of time points. To further estimate the false discovery rate (FDR), we permutated the data and assessed the background distribution of R^2^. We only consider genes with FDR < 5% as a good fitting. The segmented regression was implemented by Trendy^22^ R package.

### Gene Ontology (GO) enrichment analysis

Gene ontology (GO) enrichment analysis was performed using the R package (“allez”)^23^. The enrichment *p*-values were further adjusted by Benjamini–Hochberg (BH) multiple test correction.

### Random forest prediction model

We used the random forest regression (RFR) model to predict the retina acellular capillary density from gene expression patterns. RFR is a supervised machine learning algorithm that uses an ensemble learning method for regression. The ensemble learning method is a technique that combines predictions from multiple machine learning models to make a more accurate prediction than a single model. RFR constructs a multitude of decision trees at training time. It takes advantage of two machine-learning techniques: bagging^24^ and random feature selection. In bagging, each tree is trained on a bootstrap sample of the training data, and aggregating prediction results make predictions from trees. RFR randomly selects a subset of features to split at each node when growing a tree. To assess the prediction performance of the random forest algorithm, RFR performs a type of cross-validation in parallel with the training step by using the so-called out-of-bag (OOB) samples. Specifically, each tree is grown using a particular bootstrap sample in training. Since bootstrapping is sampling with replacement from the training data, some sequences will be ‘left out’ of the sample, while others will be repeated in the sample. The ‘left out’ sequences constitute the OOB sample. On average, each tree is grown using about 1 − e^−1^ ≅ 2/3 of the training sequences, leaving e^−1^ ≅ 1/3 as OOB. Because OOB sequences have not been used in the tree construction, one can use them to estimate the prediction performance^25^. The RF algorithm was implemented by the “randomForest” R package^26^. We first ranked the gene importance based on the permutation test to achieve the best prediction performance via a minimum number of genes. Specifically, we permutated each gene's expression patterns and calculated the prediction error changes for each permutation. Intuitively, if a gene is essential, the permutation will result in a significant decrease in the prediction performance. Hence, we can rank the gene importance based on the prediction error changes for each permutation. Then, we used a forward feature selection method (i.e., adding each gene at a time, starting with the top 3 most essential genes) to select a minimum number of genes that can achieve the best performance. The prediction performance was calculated by Pearson correlation coefficient (R) between observed and predicted acellular capillary densities. We identified that combining the top 14 genes achieved the best Pearson correlation coefficient (R). Hence, we used the gene expression patterns of the top 14 ranked genes as features to build an RFR model to predict acellular capillary density.

## Results

### The complexity of associations between acellular capillary density increasing and the joint factors of blood glucose, diet, and sex

**Figure 1:**
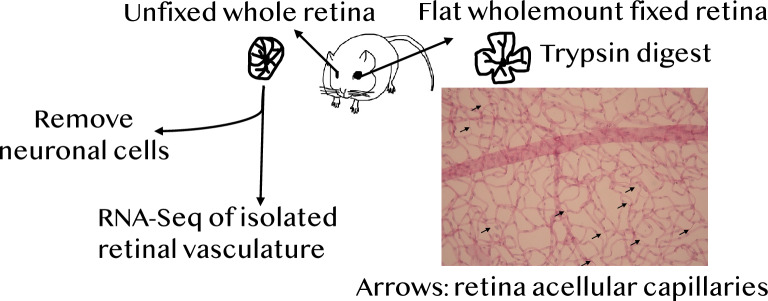
Demonstration of Nile rat retina acellular capillaries and the overall experimental design for RNA-seq. Arrows indicate acellular capillaries. Acellular capillaries are capillary-sized vessel tubes without nuclei which are considered a key symptom of the early stage of retinopathy.

**Figure 2:**
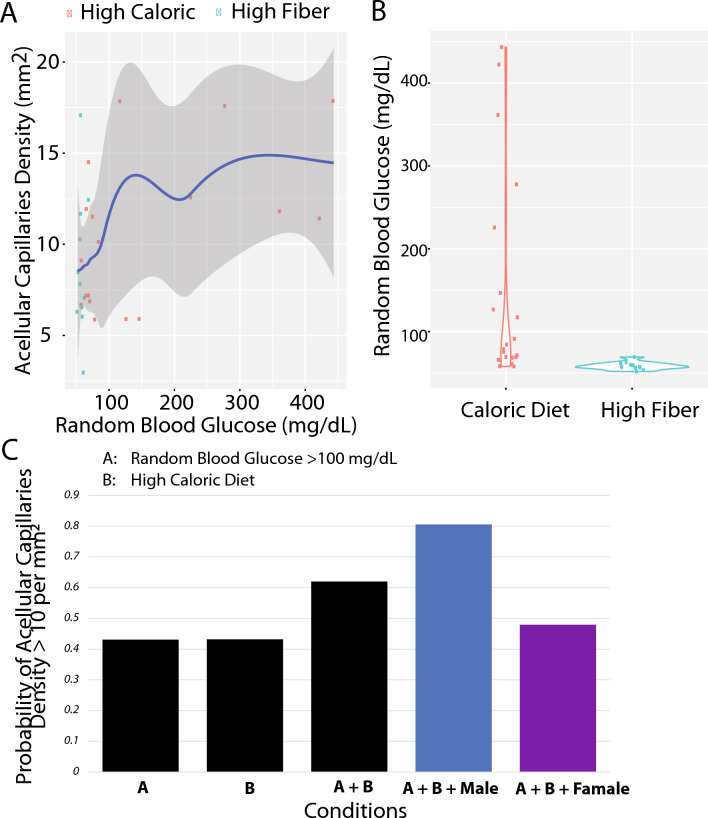
Factors associated with increased acellular capillaries density (ACD). (**A**) Random blood glucose (RBG) is moderately associated with ACD (Spearman's rank correlation coefficient Rho = 0.31, *P*-value = 0.09); (**B**) High-caloric diet is significantly associated with high random blood glucose (RBG) (Wilcoxon rank sum exact test, *P*-value = 1.38e-05); (**C**) Bayesian network modeling the conditional probability of relative higher ACD (> 10 counts per mm^2^) suggests that combined factors of high RBG, high-caloric diet, and the male will result in a twofold increase the likelihood of high ACD than any factors alone.

Increased acellular capillary density is one of the earliest detectable signs of diabetic retinopathy^9,10^. Acellular capillaries are capillary segments with no nuclei along their length, as shown in Fig. 1 (Nile rat retina acellular capillaries; indicated as arrows). Hyperglycemia is a well-known risk factor for increased acellular capillaries^9,10^. However, it is unclear how acellular capillary density is related to three relevant factors of type 2 diabetes: blood glucose levels, diet, and sex. First, we performed a Spearman's rank correlation analysis (*N* = 32; Nile rats; See Supplementary Table 1) to investigate the correlation between random blood glucose (RBG) level and acellular capillary density. As shown in Fig. 2A, the RBG is moderately associated with acellular capillary density (Spearman’s correlation coefficient Rho = 0.31, *P*-value = 0.09), indicating a positive but complex relationship between RBG and acellular capillary density, where the trend is more obvious at the pre-diabetic range of RBG. We also observed that some Nile rats with high acellular capillary density do not have hyperglycemia. Next, we investigated the role of diet. Nile rats on a high-caloric diet have higher RBG compared to Nile rats on a high-fiber diet (Fig. 2B, Wilcoxon rank sum exact test, *P*-value = 1.38e-05). In our study cohort, 61.9% of Nile rats on the high-caloric diet had not yet developed hyperglycemia, defined as RBG > 100 mg/dL^9^. Hence, we could investigate if diet could affect acellular capillary density independent of RBG. Additionally, since retinopathy can have differences between males and females, we also included sex in our acellular capillary density analysis. To understand individual factors contributing to increased acellular capillary density, we developed a Bayesian network (BN) to calculate the conditional probability of high acellular capillary density based on RBG, diet, and sex. As shown in Fig. 2C, the likelihood of a high acellular capillary density is progressively increased when factors of blood glucose level, diet, and sex are combined. For example, a Nile rat on a high-caloric diet will have a 43.07% chance of developing acellular capillary density greater than 10 counts per mm^2^. This probability increases to 62.0% if this Nile rat also has an RBG > 100 mg/dL. Furthermore, if this Nile rat is male, the possibility will further increase to 80.56%. Hence, a male Nile rat on a high-caloric diet with a high blood glucose level will have a much higher chance of presenting high acellular capillary density, a quantitative marker for early retinopathy.

### Transcriptomic profiling of retinal vasculature reveals gene expression patterns associated with elevated acellular capillary density

**Figure 3:**
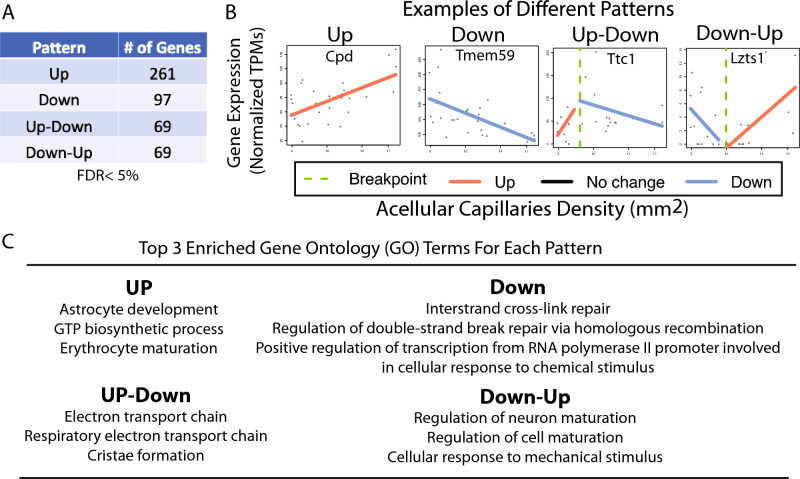
Different gene expression patterns are associated with elevated acellular capillaries density (ACD). (**A**) Segmented regression analysis identified four patterns (Up, Down, Up-Down and Down-Up) associated with elevated ACD with a False Discovery Rate (FDR) < 5%; (**B**) Examples of each pattern; (**C**) Top 3 enriched gene ontology (GO) terms for each pattern.

We hypothesized that the retinal vasculature contains molecular signals driving the early stages of retinopathy pathogenesis. To test our hypothesis, we performed RNA-seq on these whole retinal vascular tissues (See methods) of Nile rats. We have 32 Nile rat in total with a large acellular capillary density dynamic range from 2.9 to 17.9 (median: ~ 10) counts per mm^2^. However, four Nile rat samples failed to pass quality control (QC) during the RNA-seq library step and thus were excluded from the RNA-seq data analysis (Supplementary Table 1). Hence, there are 28 Nile rat retinal vascular RNA-seq data in total. The gene expression values of retinal vasculatures were quantified as normalized TPM (transcript per million) values. We performed a segmented regression analysis to identify gene signatures associated with elevated acellular capillary density (see Methods). As shown in Fig. 3, we identified four major gene expression pattern categories associated with elevated ACD: Up (gene expression up-regulated); Down (gene expression Down-regulated); Up-Down (gene expression first goes up and then followed by down-regulation); Down-Up (gene expression first goes down and then followed by up-regulation). Examples of each pattern can be seen in Fig. 3B. Interestingly, the gene ontology (GO) term (Astrocyte development) is the top one enriched in genes with the Up pattern. Studies have shown that glucose tightly controls the morphological functions of astrocytes^27^, suggesting a direct or indirect impact of hyperglycemia on retinal vasculature transcriptomic changes. The down-regulated genes (negatively correlated with acellular capillary density) are enriched in DNA and cellular repair/response-related functions, such as inter-strand cross-link repair. It has been known that macular (a light-sensitive layer in the center of the retina) degeneration is tightly associated with impaired retina DNA damage and repair mechanism^28,29^, which is characterized by the sensitivity of retinal pigment epithelial (RPE) to blue and UV lights exposure. Interestingly, the impaired DNA damage repair mechanism is observed in both macular degeneration and early retinopathy. The Up-Down and Down-Up patterns are associated with a wide range of biological functions (See Supplementary Table 2), such as electron transport chain (Up-Down pattern) and regulation of neuron maturation (Down-Up) patterns, indicating the complexity of dynamic transcriptomic changes in response to elevated acellular capillary density. The top enriched gene ontology terms for each pattern are shown in Fig. 3C.

### Acellular capillary density can be quantitively imputed by the expression patterns of fourteen genes

**Figure 4:**
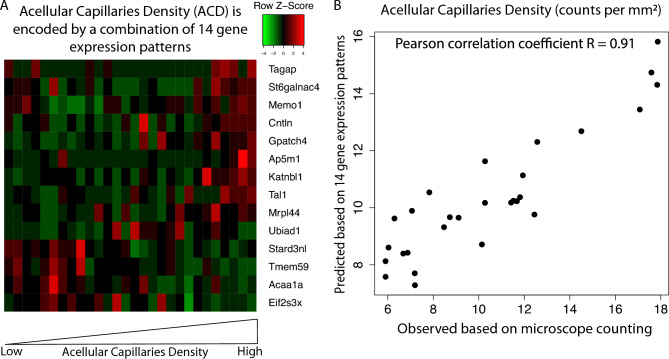
Random forest regression model to predict acellular capillaries density. (**A**) Random forest regression model identified a combination of 14 genes that can quantitively predict acellular capillaries density. (**B**) The predicted acellular capillaries density is highly correlated with the observed acellular capillaries density.

We hypothesize that the pathological characteristics of a dysfunctional retina are encoded in their transcriptomic patterns. Thus, subtle changes associated with the early progression of diabetic retinopathy, as quantified by acellular capillary density, can be inferred from a combination of gene expression patterns. To identify a minimum number of genes that can be used to impute acellular capillary density, we develop a random forest regression (RFR) model. We ranked the gene importance based on the prediction performance changing by permutation test (i.e., permutating gene expression patterns for each gene). Then we used a forward feature selection method (i.e., adding each gene at a time) to select a minimum number of genes that can achieve the best performance (See Methods). As shown in Fig. 4A, we identified a combination of 14 genes that can predict acellular capillary density with high accuracy (Pearson correlation coefficient R = 0.91; Fig. 4B). It suggests that acellular capillary density is uniquely encoded in gene expression patterns of these 14 genes, and a machine-learning regression model can decode the transcriptomic signals associated with this early retinopathy feature.

### Reversal transcriptome identifies putative acellular capillary inhibitor candidates

**Figure 5:**
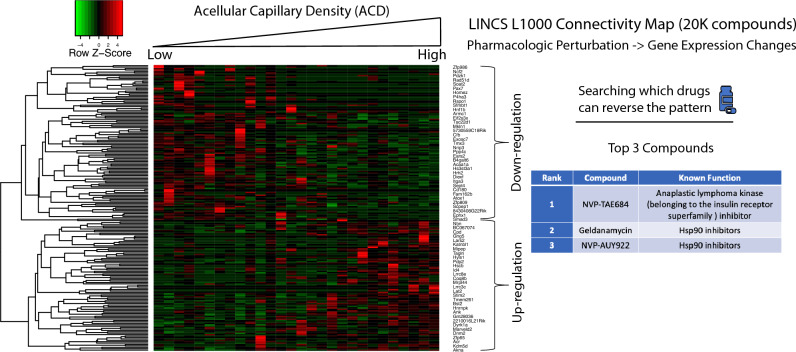
Identification of putative candidates that can reduce acellular capillary density via reverse transcriptome. The heatmap shows genes with |Rho|> 0.4 between gene expression values and acellular capillaries density (ACD). Searching LINCS L1000 chemical compound perturbated gene expression database identified NVP-TAE684, geldanamycin, and NVP-AUY922 as the top three candidates.

Currently, there are no effective ways to treat or reverse early retinopathy. Emerging evidence suggests that the reversal of disease gene expression patterns correlates well with drug efficacy and reveals therapeutic targets^30^. Specifically, if a small molecule or compound can reverse or partially reverse disease marker gene expression patterns, it can be a potential disease treatment drug. Given that elevated acellular capillary density is one of the earliest quantitative features of diabetic retinopathy, we hypothesize that if a compound can reverse or partially reverse the acellular capillary density-associated transcriptome, it could be a putative drug candidate to alter the progression toward vision loss. To this end, we identified genes that are positively or negatively correlated with acellular capillary density levels via Spearman’s rank correlation analysis (Absolute Correlation coefficient (Rho) > 0.4; Fig. 5). Absolute Rho values greater than 0.4 are generally considered to have correlated patterns^31^. We then searched LINCS L1000 chemical compound perturbated gene expression database^32^ to find drugs that could potentially reverse this pattern (i.e., a compound that can down-regulate the gene expression for acellular capillary density positively correlated genes while up-regulating the gene expression for acellular capillary density negatively correlated genes). We found NVP-TAE684, geldanamycin, and NVP-AUY922 to be the top three compounds identified to potentially reverse the retinal vascular phenotype associated with high acellular capillary density (Fig. 5). NVP-TAE684 is an anaplastic lymphoma kinase (ALK) inhibitor. ALK is a receptor tyrosine kinase belonging to the insulin receptor superfamily sharing a high degree of homology with leukocyte tyrosine kinase (LTK)^33^. Both geldanamycin and NVP-AUY922 are Hsp90 inhibitors^11,12^. Interestingly, studies have shown that a short-time treatment of Hsp90 inhibitor can stimulate a retina stress response with molecular chaperone expression, preventing retinal degeneration in models of retinitis pigmentosa and age-related macular degeneration^13^. Although no study shows that Hsp90 inhibitors can be used to treat early retinopathy, there is evidence that Hsp90 inhibitors, such as AUY922, can protect and repair microvascular endothelial cells related to endothelial barrier dysfunction in a different biological context (e.g., human lung^34^). Retina endothelial dysfunction is a significant complication that drives diabetic retinopathy^35,36^. Hence, it is likely that Hsp90 inhibitors (geldanamycin and NVP-AUY922) can potentially treat or protect early retinopathy via modulating microvascular endothelial cells.

To further evaluate our predicted drugs, we performed a text-mining approach. We listed a set of retinopathy-associated anatomical features: Microaneurysm, Pericyte loss, Dot haemorrhages, Intraretinal haemorrhages, Hard exudates, Cotton wool spots, Neovascularization, Venous beading, Ischemia. We then used a text mining tool (KinderMiner: https://www.kinderminer.org/login) to calculate whether the predicted compounds (NVP-TAE684, geldanamycin and NVP-AUY922) are statistically enriched in any of the retinopathy-associated anatomical features as listed above via scanning 34,006,217 PubMed articles). We found geldanamycin is statistically enriched in “Neovascularization term” (*P*-value = 0.001) and “Ischemia” term (*P*-value = 8.7E-10). Since both geldanamycin and NVP-AUY922 are Hsp90 inhibitors, we further calculated the enrichment *P*-values of the term “Hsp90 inhibitors” in retinopathy-associated anatomical features based on KinderMiner tool. The term “Hsp90 inhibitors” is statistically enriched in “Neovascularization term” (*P*-value = 0.02) and “Ischemia” term (*P*-value = 6.6E-5). This indirectly supported that our predicted Hsp90 inhibitors (geldanamycin and NVP-AUY922) could potentially attenuate or mitigate prodromal diabetic retinopathy.

## Discussion

Diabetic retinopathy is a progressive disease that will eventually lead to vision loss. Although current technological advances have led to easy detection of early retinopathy prior to any vision impairment, strategies to address early retinopathy and prevent vision loss are lacking. Human tissues presenting early retinopathy are scarcely available. Hence animal models are needed. Diabetic Nile rats develop retinal lesions similar to human diabetic retinopathy associated with vision loss^9^, which may arise from shared molecular mechanisms.

In our previous study, we found that acellular capillary density is a quantitative feature associated with risk factors of diabetic retinopathy at least in the Nile rat model. The acellular capillary density is apparent earlier with a large dynamic range compared to other vascular characteristic changes such as pericyte loss (small dynamic range) and neuronal lesions (apparent later) in the Nile rat. We hypothesize that molecular signatures initiating early retinopathy are encoded in the retinal vasculature and therefore are also associated with acellular capillary density. We developed a machine learning model via gene expression patterns of fourteen genes that can quantitively predict the acellular capillary density. Since acellular capillary density is a quantitative feature associated with early diabetic retinopathy, our model can be used as a retinopathy early progression “transcriptomic clock” measuring the severity or the progression of early retinopathy. However, we acknowledge that this model was based on Nile rat diabetic retinopathy data. Comparisons with other animal models and humans are needed to validate the gene expression pattern and if this prediction model is consistent across species.

Emerging evidence suggests that reversal of disease gene expression patterns correlates well with drug efficacy^30^. Hence, we hypothesize that if a small molecule or a drug can reverse the majority or a significant fraction of the DR-driven gene expression changes, it can be a putative DR treatment drug candidate. We used acellular capillary density-associated up- and down-regulated gene signatures to identify drugs that can potentially reverse or partially reverse such patterns via searching LINCS L1000 chemical compound perturbated gene expression database^32^. The fundamental basis for this approach assumes that targeting early vascular changes is the key to blocking early retinopathy. This assumption is based on our prior study that the elevated acellular capillary density was a reliable quantitative trait present in the initial development of retinopathy preceding additional vascular dysfunction in pericytes and endothelial cells^9^. Among the top three predicted drugs, geldanamycin, and NVP-AUY922 are Hsp90 inhibitors^11,12^. Some studies have shown that Hsp90 inhibitors can protect and repair microvascular endothelial cells related to endothelial barrier dysfunction but in a different biological context: human lungs^34^. Although we need more in vivo studies in the future to support our findings, we provide a data-driven approach to re-purpose treatment drugs, which can largely shorten the traditional drug discovery path. However, we also acknowledge that in vivo and in vitro studies are needed to validate these re-purposed drugs further.

## Conclusions

Our study not only gives transcriptomic insights into the pathogenesis of early-stage retinopathy but also provides a novel data-driven approach to prioritize and re-purpose treatment drugs.

### Supplementary Information

Supplementary Legends.

Supplementary Table 1.

Supplementary Table 2.

## Data Availability

We have submitted the RNA-seq data and the gene expression data (TPMs and Mapping counts) to GEO (accession number: GSE220672).

## Abbreviations

DR: Diabetic retinopathy
BN: Bayesian network
RBG: Random blood glucose
ACD: Acellular capillary density
GO: Gene ontology
RFR: Random forest regression
OOB: Out-of-bag
RPE: Retinal pigment epithelial
