# Supplementary material for: Transcriptomic clock predicts vascular changes of prodromal diabetic retinopathy
Source: Sci Rep. 2023 Aug 10;13:12968. doi: 10.1038/s41598-023-40328-w (PMC10415264; doi:10.1038/s41598-023-40328-w)
Supplement: Supplementary file 1 — Supplementary Legends. [file 41598_2023_40328_MOESM1_ESM.docx]

## List of supplementary tables

**Supplementary Table 1:** Detailed Nile rats information. There are 32 Nile rats in total in this study. Four Nile rat samples were excluded from the RNA-seq data analysis due to technical issues (failed to pass quality control during RNA-seq library preparation step). Therefore, there were twenty-eight samples included in the RNA-seq data analysis.

**Supplementary Table 2**: Enriched gene ontology (GO) terms (Benjamini-Hochberg adjusted p-values < 0.05) for four major gene expression pattern categories associated with elevated ACD: Up (gene expression up-regulated); Down (gene expression Down-regulated); Up-Down (gene expression first goes up and then followed by down-regulation); Down-Up (gene expression first goes down and then followed by up-regulation).
